# Influence of Human Activity on Radon Concentration, Indoor Air Quality, and Thermal Comfort in Small Office Spaces

**DOI:** 10.3390/s24154949

**Published:** 2024-07-30

**Authors:** Nina Szczepanik-Scislo, Dominik Grządziel, Jadwiga Mazur, Krzysztof Kozak, Jacek Schnotale

**Affiliations:** 1 Faculty of Environmental Engineering and Energy, Cracow University of Technology, Warszawska 24, 31-155 Cracow, Poland; jacek.schnotale@pk.edu.pl; 2 CERN, European Organization for Nuclear Research, Esplanade des Particules 1, 1211 Meyrin, Switzerland; 3 Institute of Nuclear Physics Polish Academy of Sciences, Radzikowskiego152, 31-342 Cracow, Poland; dominik.grzadziel@ifj.edu.pl (D.G.); jadwiga.mazur@ifj.edu.pl (J.M.); krzysztof.kozak@ifj.edu.pl (K.K.)

**Keywords:** indoor air quality, radon monitoring, CO_2_ concentration, thermal comfort

## Abstract

This article focuses on the influence of occupants on the concentration of contaminants (radon Rn-222 and CO_2_) as well as the thermal comfort parameters. A series of sensors were placed to measure the concentration of the contaminants, temperature, and relative humidity in the test room at the Institute of Nuclear Physics PAN in Krakow (IFJ PAN), Poland. The test room is an office that is typical of the offices used in the facility. The occupants that used the space kept a detailed diary of their entry, exit, and number of people entering. The results showed that the accumulation of contaminants in such spaces may be severe and risks the health and safety of occupants. The accumulation of CO_2_ was extremely noticeable and did not diminish to the background level between the re-entry of the occupants to the office. The same was consistent for the radon concentration. The study shows how ventilation methods and small changes in occupant work strategy may influence the contaminant concentration within a test zone.

## 1. Introduction

People spend most of their time inside buildings, where levels of harmful pollutants are often significantly higher than outdoors. Prolonged exposure to these elevated levels of such pollutants can lead to illness and disrupt the comfort of occupants in the workplace. Factors such as ventilation methods, occupant contribution, and faulty equipment significantly influence air quality [1,2,3]. These contaminants can include commonly found pollutants such as carbon dioxide (CO_2_), as it may have a high background level and humans are large contributors to its concentration in the indoor air [4]. They can also be such contaminants as radioactive gasses that can be found in the surrounding environment. 

An example of a radioactive pollutant is radon gas (Rn-222) and its decay products, which are the primary source of ionising radiation inside buildings. Indoor radon concentrations mainly depend on the geological composition of the ground where the building is situated [5,6]. Radon migrates to the buildings from the ground through all types of foundation leakages. Construction materials used, especially if they contain ashes and furnace slag, are also a source of radon indoors [7]. An additional indoor radon source can be Ground Heat Exchangers (GHEs), which are increasingly used in private homes and institutions. However, both homeowners and GHE producers are usually not aware of the possible danger of radon. In some cases of open GHEs, indoor radon concentration can increase significantly during GHE operation. The influence of inhabitants’ activity and habits on indoor radon cannot also be neglected. Radon and its progeny emit highly energetic alpha radiation that may be harmful to human health.

Epidemiological studies conducted around the world have confirmed the impact of the presence of radon in homes on the increased risk of lung cancer. It is estimated that 3% to 14% of lung cancers worldwide are caused by radon. Radon has been recognised as the second carcinogenic factor for lung cancer, after tobacco smoking. The impact of radon on the development of lung cancer is greater for smokers (current and former) than for non-smokers. It is assumed that, for non-smokers, radon is the first carcinogenic factor.

There is no minimum threshold of radon activity concentration defined at which there is no risk of lung cancer. Even very low concentrations of this gas contribute to an increase in the risk of disease. It is believed that initial cancer induction may occur even as a result of damage to a single cell. Hence, even a single alpha particle originating from one radon atom can cause an undesirable mutation. Most radon-related lung cancers are caused by exposure to low and medium concentrations of radon, and rarely to high levels [8]. Therefore, research on indoor radon is an essential component of indoor air quality (IAQ) assurance [8].

Carbon dioxide is often also used as a component to assess the IAQ along with the thermal comfort parameters such as temperature and relative humidity (RH). Despite many regulations, the problem of high contaminant concentration and lack of thermal comfort has been proven in many buildings [9,10,11,12,13]. This problem has occurred in households, sports facilities [14,15], schools [16,17,18,19], kindergartens [20], office environments [21], etc., which means that it is a concern for the entire building sector [1,22].

This study focused on the indoor air quality inside small office spaces with natural ventilation. Both radon and CO_2_ were measured as IAQ indicators, and the temperature and relative humidity were thermal comfort factors. A radon source was introduced in the tested area to see how occupant activity influenced its concentration, recreating a scenario in which the radon would leak in from the external environment. The only indoor source of CO_2_ was the occupants themselves.

The sensor technology used in the study measured the impact of occupants on small spaces when there is additional contamination. While it is known that human behaviour influences CO_2_ concentration due to humans being a source of this contaminant, it is also important to determine if different pollutant concentrations are not influenced by human behaviour.

Radon, a radioactive gas that emanates from certain rock and soil types, is present in environments like underground laboratories. Radon can seep into these spaces from the ground, independent of any human action. Nuclear institutions such as the Institute of Nuclear Physics, the Polish Academy of Sciences in Krakow, and the European Organisation for Nuclear Research (CERN) have underground laboratories. In these facilities, radon has been a source of additional radiation.

The study aimed to explore the influence of occupants on CO_2_ levels, which is closely linked to their occupancy and work schedules, to determine if radon concentrations, whose source is independent of human behaviour, are also affected. By comparing the levels of these two contaminants, it was reviewed how occupant schedules impact two pollutant sources that are independent of one another and whether one pollutant might influence the levels of the other.

To achieve this, sensors were deployed to continuously monitor both CO_2_ and radon levels in the specified environments. Data were collected and analysed to recognise patterns correlating with occupancy. This approach allowed to identify the extent to which human presence and activity contribute to variations in CO_2_ and whether radon levels exhibit any fluctuations corresponding with human schedules.

The outdoor CO_2_ concentration was also measured but did not influence the indoor conditions (average of 450 ppm with a maximum of 480 ppm). The study aimed to investigate how occupant activity could impact contaminant concentration in the given scenario.

## 2. Materials and Methods

**Figure 1:**
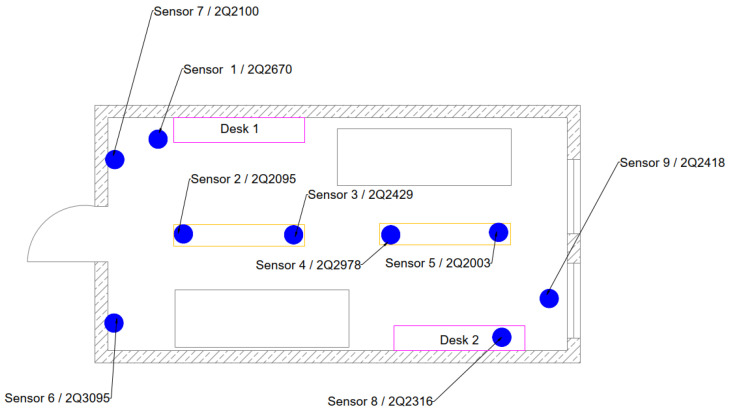
Arrangement of measurement points in the controlled office.

The study aimed to assess the influence of occupants on contaminant migration, air quality, and thermal comfort. A series of dynamic measurements were performed using devices in nine points that measured radon Rn-222 activity concentration (RAC), carbon dioxide concentration, air temperature, and relative humidity in a controlled office with 2.8 m × 5.4 m dimensions. These devices were placed uniformly in the entire volume of the office. Additionally, two active radon monitors, AlphaGUARD (Saphymo, Germany) PQ2000 and PQ2000 PRO, were placed near measuring points on the desk near the entrance and on the window (near sensors 1 and 8 in Figure 1). The source of radon was also placed at the office (on the desk near the entrance). It was a PYLON RN-1025 (manufactured by PYLON ELECTRONICS INC., Ottawa, ON, Canada) source with radium Ra-226 activity of 21.6 kBq, enabling the production of radon at the rate of 2.71 Bq/min. Radon gas was released into the indoor air continuously during the experiment. The layout of the device locations is shown in Figure 1.

The sensors were planted at different locations and heights in the test office. Sensors 6 and 7 were located close to the ceiling, next to the exhaust grilles, at a height of approximately 3 m above the floor. They were placed to see how the exhaust air was contaminated. The exhaust air is the uniformly mixed air from the room, so it was possible to use these measurements to reference any local peaks of contaminant contraction if they occurred. Sensors 1 and 8 were located on two desks: in the corner of the room near the entrance (sensor 1—desk 1) and close to the window on the opposite side of the room (sensor 8—desk 2). Sensors 2, 3, 4, and 5 were hung approximately 2 m above the floor to see the layout in the room close to the occupants but to not interfere with their work. Sensor 9 was located on the window to see if the infiltration process of the natural ventilation system influenced the contraction where the fresh air should be coming through using the leakage in the building envelope.

The PYLON radon source was located on desk 1. Active radon monitors AlphaGUARD were located close to the sensors: AG-1 on desk 1 close to sensor 3, and AG-2 on the window, close to sensor 5.

The test room had a natural ventilation system with two exhaust grilles mounted near the door. The door to the room was kept closed and only opened when the occupants were entering or leaving.

During the measurements, the outdoor level of CO_2_ was measured and averaged at 450 ppm.

Each measurement point consisted of two sensors. The first sensor measured the radon activity concentration, and the second one measured the CO_2_ concentration, temperature, and relative humidity. Radon activity concentration was measured using a passive method with solid-state nuclear track detectors (SSNTD) type CR-39. This method provided only one averaged value of radon activity concentration over the entire measurement period. The active sensors for registering the concentration of CO_2_, air temperature, and humidity were measured in 10 min time steps, and all the results were recorded. Two active radon monitors (AlphaGUARD) continuously measured radon activity concentration in 10 min time steps. The operating modes of these active devices have been time-synchronised.

The passive radon detector consists of a plastic cover (diffusion chamber), inside which the CR-39 foil is placed, which is the actual radon detector. Air with radon present in it penetrates inside the diffusion chamber through the aperture. Alpha particles emitted by radon cause microscopic damage in the CR-39 film. After the exposure, CR-39 foil is removed from the diffusion chamber, then etched in a 25% solution of sodium hydroxide (NaOH) and alpha traces, seen as dark spots on CR-39 foil, are counted. Knowing the calibration factor, it is possible to determine the average radon activity concentration during the detector exposure based on the density of the traces.

The professional radon monitor (AlphaGUARD) is an active device that measures radon activity concentration in real time. It incorporates a pulse-counting ionisation chamber based on alpha spectroscopy. Having optimal chamber geometry and intelligent signal evaluation, AlphaGUARD is suitable for the continuous monitoring of a wide range of radon activity concentrations. Long-term stable calibration factor (guaranteed for five years), calibration traceable to different national standards, and inbuilt quality assurance system allow AlphaGUARD to serve as a reference device.

Nine sensors (calibrated by the manufacturer) were used to measure real-time carbon dioxide concentrations, relative air humidity, and air temperature.

The temperature, relative humidity and carbon dioxide concentration were measured using the wireless transmitter iBros T/RH/CO_2_, operating on the IEEE 802.15.4 [23] protocol with a transmission frequency of 2.4 GHz. The base station EE242 can coordinate a network of up to 500 WiFi-connected devices. Additionally, the base station connects to a PC for real-time data evaluation or from cloud-stored files. CO_2_ measurement utilises dual-wavelength non-dispersive infrared (NDIR) technology. This spectrometer is non-dispersive because the broadband light is not separated into a narrow spectrum by dispersive elements like a monochromator. Infrared (IR) sensors typically include an IR source (lamp), a sample chamber, a light filter, and an infrared detector. The sample chamber directs IR light toward the detector, while a parallel chamber contains an enclosed reference gas, usually nitrogen. The detector measures the attenuation of specific wavelengths caused by gas absorption in the sample chamber, based on the Beer–Lambert law, to determine the gas concentration. Detectors have optical filters that block all light except that absorbed by the selected gas molecules [24].

The measurement range and accuracy of the whole equipment are shown in Table 1.

The measurements’ duration was one month, from 22 January to 21 February. They were performed at the Institute of Nuclear Physics, Polish Academy of Sciences in Krakow (IFJ PAN), Poland.

During the measurement period, the users of the controlled office prepared a detailed diary of when they entered the room, how many occupants there were, and for how long it was occupied. The test room served as an office for one of the research staff, who moved between the laboratory facility and the test office when needed. Only this occupant usually occupied the room during the test period. However, a maximum of 3 occupants were noted in the diary received due to the collaboration between a group of researchers. The windows stayed closed during the entire experiment. The occupant that was in the office most often used the desk near sensor 1 for writing research papers. This occupant’s activity was considered light as he sat or stood at the desk. When more than one occupant was in the test room, it was to review a research paper while sitting at the desk near sensor 1.

The only indoor source of CO_2_ was human respiration. The contaminant concentration will show how the ventilation system removed the CO_2_ and if it efficiently maintained proper hygienic levels. At low levels, carbon dioxide is a nontoxic gas. However, high levels of CO_2_ can be dangerous to human health. In the past, ASHRAE standards (62-1989) [25] considered 1000 ppm (parts per million) as the acceptable maximum concentration for indoor carbon dioxide and a minimum ventilation rate of 8 l/p/s. More recent standards (ASHRAE 62-1999, 62-2001, and 62-2004) [26,27,28] recommend that indoor–outdoor differential concentration should not exceed 700 ppm. The reason for such limitations is that concentrations of CO_2_ above 1000 ppm can lead to headaches, dizziness, and other symptoms of CO_2_ toxicity and can be dangerous in enclosed spaces where ventilation is limited [29,30,31]. In extreme cases, such high levels of the contaminant can lead to unconsciousness and death. Maintaining good air quality and ventilation is essential, especially in enclosed spaces where CO_2_ levels can quickly build [32,33,34,35,36].

Directive 2013/59/EURATOM of the Council of the European Union introduced exposure to radon (Rn-222) to the group of internal exposures resulting from ionising radiation. The directive’s recommendations oblige European Union member states to introduce a reference level for indoor radon activity concentration. This level, expressed as the value of the average annual radon activity concentration, is 300 Bq/m^3^ and is the same for both residential buildings and workplaces. Exceeding this level will require taking appropriate measures to limit exposure to radon. Exposure to radon is equivalent to exposure to its decay products.

These recommendations were implemented in national regulations in September 2019 by the Atomic Law Act. In 2021, the Announcement of the Minister of Health established a national action plan in the case of long-term threats resulting from exposure to radon in residential buildings and workplaces, the so-called “radon action plan”.

Parameters such as temperature and relative humidity were recorded when considering thermal comfort. The room was heated by a central heating system with water-filled radiators that should maintain a steady temperature. This was designed to provide the occupants with an optimal sensation of warmth [37,38]. However, as the study was conducted during the winter months, there is a risk that the air may have been too dry for occupants, as this is a common occurrence [39,40].

## 3. Results and Discussion

### 3.1. Radon Activity Concentration

Radon activity concentration was measured in the entire test room at nine points using a passive method. In addition, one CR-39 radon detector was placed outside the window and measured radon gas concentration in the atmospheric air. The RAC in the atmospheric air was 66 ± 22 Bq/m^3^.

**Figure 2:**
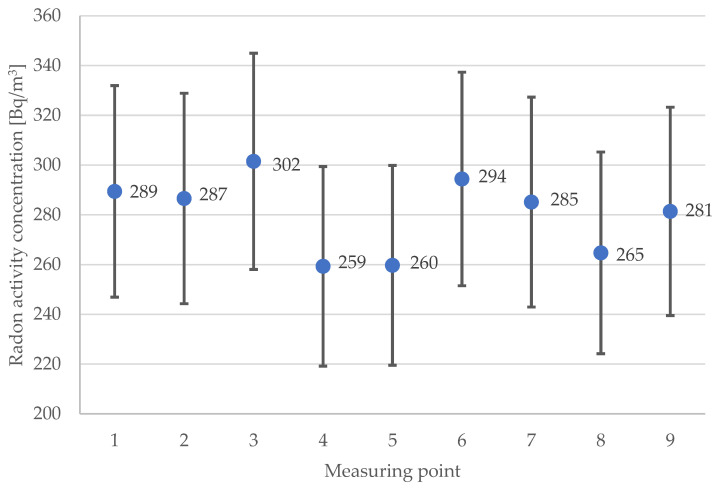
Distribution of radon activity concentration (RAC) in the measuring points.

The sensors and radon detectors were placed evenly throughout the room at different heights. Therefore, it was possible to study the room’s homogeneity of all recorded parameters (radon activity concentration—RAC, CO_2_, temperature, humidity). The results of RAC are presented in the distribution graph (Figure 2), which show that the concentration of radon activity throughout the room was uniform. The statistical Dixon test showed that there are no outliers within the results. The mean value of radon activity concentrations amounted to 280 Bq/m^3^, ranging from 259 Bq/m^3^ to 302 Bq/m^3^. The median value was 285 Bq/m^3^, and the standard deviation (SD) was 15 Bq/m^3^.

**Figure 3:**
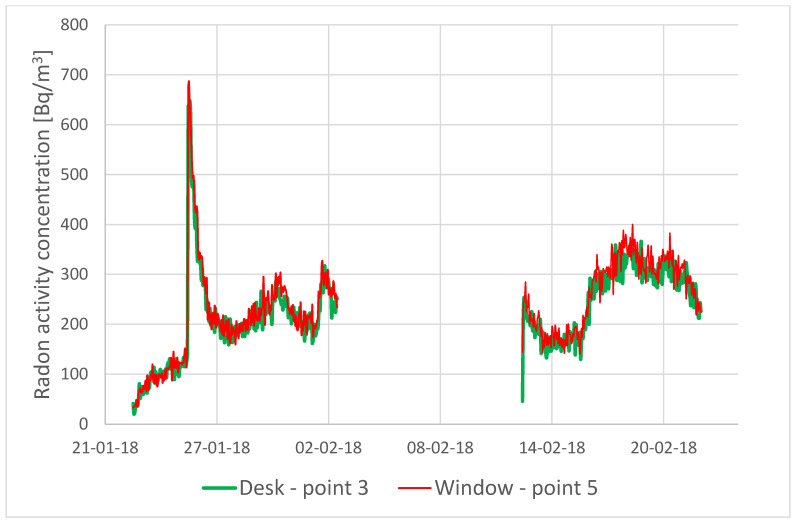
Radon activity concentration course at 2 points: desk and window.

Also, the course of radon activity concentrations in two separate points (3 and 5), recorded with 2 AlphaGUARD monitors, was examined. This course is basically identical at both points, even though point 3 was located close to the radon source and point 5 was close to the window (where we could expect the highest effect of ventilation processes due to air infiltration)—Figure 3.

The measurements proved that radon disperses uniformly in the room, even despite its weight. Although radon is significantly heavier than air—approximately 7.6 times denser—natural diffusion and air movement allowed it to spread evenly throughout the space due to the small size of the room.

### 3.2. Carbon Dioxide

**Figure 4:**
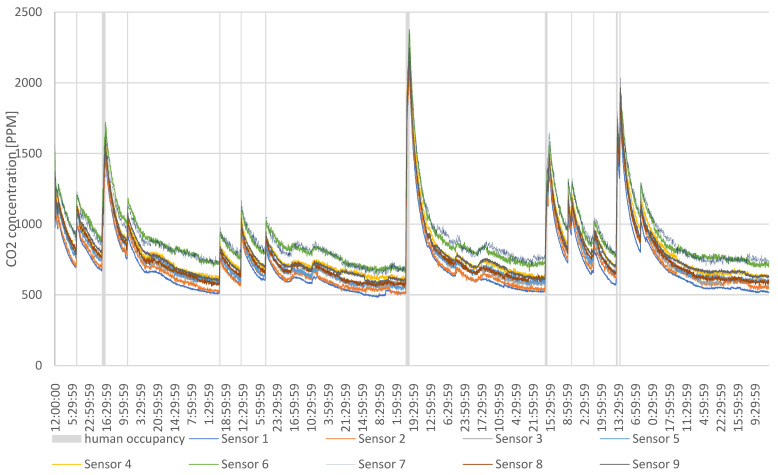
CO_2_ concentration.

During the measurements, the outdoor level of CO_2_ was measured and averaged at 450 ppm. The only indoor source of CO_2_ was from occupants entering the test room. To properly assess how they influenced the contaminant concentration, they noted the time they entered it as well as how many occupants entered. The results of the measurements are shown in Figure 4.

The figure shows that the CO_2_ concentration is affected by the occupants’ schedules. In Figure 4, the grey bars show the entrance and duration of occupants’ stay. This proves that the concentration of carbon dioxide rises very rapidly when the occupants enter the test room. Not only does it remain high during their stay, but it remains increased for hours after the occupants have left. The highest contraction was when three people entered the room for a period of 4 h. The CO_2_ concentration spiked to the level of over 2000 ppm and only returned to a safe level of 1000 ppm the next day, around 23 h after the occupants left the room.

Similar cases occurred when occupants entered the room on consecutive days. Even though they were in the room for a period of only 3 h, the carbon dioxide levels did not drop below the level of 1000 ppm before they entered again. Moreover, if the concentration dropped below 1000 ppm before the occupants re-entered, the CO_2_ level was still above 700 ppm. This means that, even though the room was not occupied for over 12 h, the contaminant levels did not drop to the background level of 450 ppm.

The results indicate that the ventilation system was not effectively removing contaminants. If occupants were to occupy such spaces constantly, the level of CO_2_ would probably never be adequate for human occupancy.

### 3.3. Thermal Comfort

Thermal comfort refers to the state of mind that expresses satisfaction with the thermal environment. It is a subjective perception of how comfortable or uncomfortable an individual feels with their surroundings’ temperature, humidity, air movement, and radiant heat.

The study measured two thermal comfort parameters: temperature and relative humidity. Although there are more complex indicators to determine thermal comfort, these two can show if there are any significant problems in the occupied space concerning the comfort of occupants [41].

Temperature and relative humidity are some vital components of indoor air quality assessment due to their direct influence on health, comfort, and productivity. Effective control and monitoring of these parameters can mitigate the adverse effects of poor IAQ, contributing to healthier and more comfortable indoor environments. They can have an impact on the health and comfort of occupants [42]. Poor IAQ, often resulting from inadequate temperature and humidity control, is a key contributor to sick building syndrome (SBS). This syndrome leads to symptoms like headaches, dizziness, and respiratory problems among occupants [43,44].

Studies have shown that thermal comfort and optimal humidity levels enhance cognitive function and productivity. For instance, in educational settings, students perform better in environments with well-regulated temperatures and humidity [43,44].

For the indoor environment to be suitable for occupants, the temperature should be 18–28 °C, while the relative humidity should be between 30 and 70% [41]. Maintaining a comfortable thermal environment is essential for occupant well-being, productivity, and health [45].

**Figure 5:**
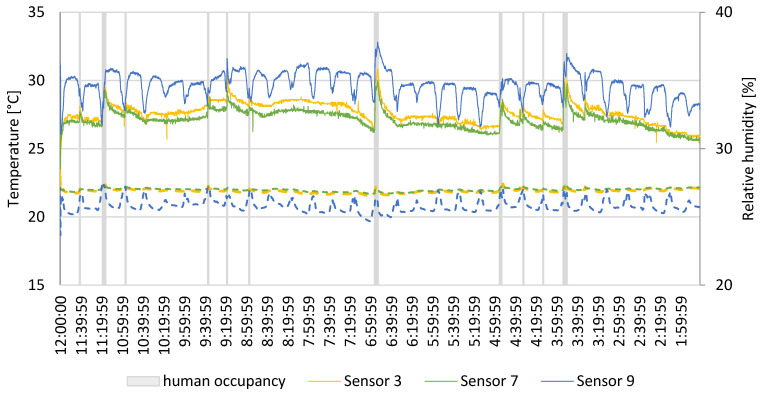
Thermal comfort parameters—solid line: relative humidity; dashed line: temperature.

The results of the temperature and relative humidity measurements are shown in Figure 5. Only three sensors (sensors 3, 7 and 9) are shown, as they represent the maximum, average, and minimum values recorded during the measurement process. The figure shows that the temperatures in the centre of the room and near the exhaust (sensors 3 and 7) are stable throughout the study. The presence of occupants only slightly changed the temperature but did not significantly affect the sensation of thermal comfort, and the temperature only rose by 0.5 °C or less. Sensor 9, however, shows both large temperature and relative humidity fluctuations. This is due to its location. It was placed near the windows, and the temperature rose during the day and then lowered when the sun set. The opposite happens to the relative humidity in this area, as it lowers when the area becomes warmer. The occupant’s effect on the relative humidity is more visible. However, it does not drastically change the humidity, leaving the air below the 35% mark, meaning it would be dry and uncomfortable for occupants.

When the heating system supplied from a city district heating network was turned on, the temperature during the day was uniform. The sensors closer to the radiators did not show a significant increase in temperature compared to the rest of the sensors. The difference in the buoyancy of the air, which is connected to the hot air close to the radiator rise, helped with the uniformisation of the concentrations and temperatures of the air inside the test office. As shown in the figures in the previous sections and in Figure 5, the presence of the occupants entering and leaving the room had the highest impact on indoor air conditions.

### 3.4. Correlation between Human Activity and Radon Activity Distribution

**Figure 6:**
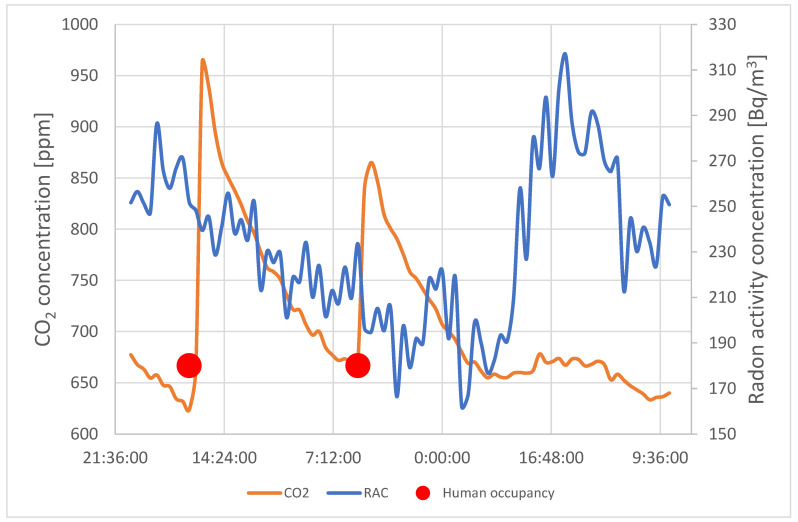
RAC and CO_2_ concentration course comparison—desk, point 3.

**Figure 7:**
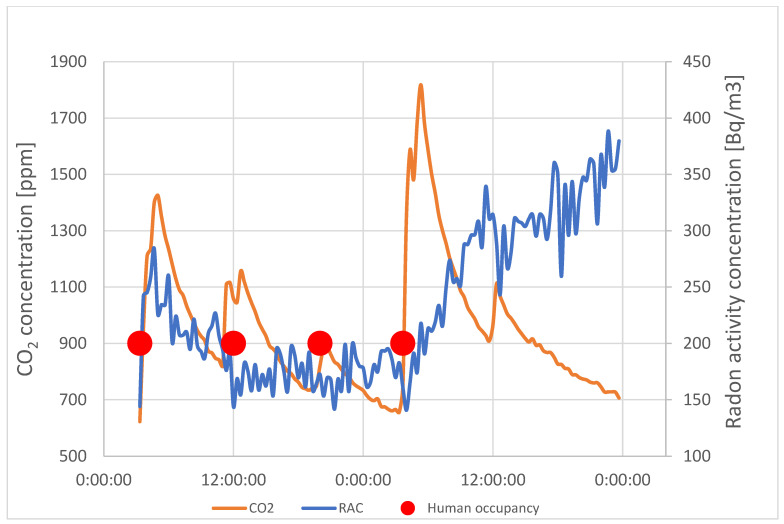
RAC and CO_2_ concentration course comparison—window, point 5.

Figure 6 and Figure 7 present the comparison of RAC and CO_2_ concentration courses at point 3 and point 5. RAC was measured at two points (AG-1, AG-2) by AlphaGUARD monitors. Monitor AG-1 was located on the desk near the radon source (RN) in the vicinity of point 3. Monitor AG-2 was located on the window in the vicinity of point 5. The distance between point 3 and point 5 was about 2 m.

The increase in CO_2_ concentration in both points is caused by users entering the room. At this moment, the concentration of radon activity decreases. Opening the door causes a movement of air that removes radon gas from the air in the test room. Due to the presence of a radon source in the room, the RAC in the room was higher than in the corridor. Opening the door caused an escape of radon gas from the place with its higher concentration. When the users left the room, the concentration of the CO_2_ gas decreased and returned to its previous value. At the same time, the activity concentration of radon in a closed, unventilated room was increasing.

However, the occupants’ presence and the concentration of CO_2_ itself did not affect the radon concentration while the occupants were in the room. This means that the movement of air inside the room due to the movement of people did not affect the contaminant distribution.

## 4. Conclusions

The study focused on the influence of occupants on indoor air quality in an office space. Over a period of one month, the concentrations of carbon dioxide (CO_2_), thermal factors, and radon (Rn-222) were meticulously measured.

The presence of occupants had a notable impact on the concentration of CO_2_ in the air. CO_2_ levels rose quickly when occupants were present, and it took approximately 23 h to return to the ambient levels of the contaminant. When the office was occupied for consecutive days, the CO_2_ concentration did not have sufficient time to normalise before occupants re-entered, leading to a cumulative increase in CO_2_ levels.

Conversely, the presence of occupants had a different effect on radon concentration. When the office door was opened, fresh air entered the space, reducing the radon levels. This occurred because the radon concentration in the corridors was lower than inside the office, allowing radon to dissipate. Once the occupants were inside the room with the door closed, their presence did not significantly influence the radon levels. This was likely due to the room’s size and the ventilation system, which did not effectively remove radon, allowing it to accumulate despite the occupants’ presence being a less influential factor.

The results for both contaminants highlight the critical role of the ventilation system in influencing indoor air quality. Despite the presence of two exhaust vents, there was a significant accumulation of both CO_2_ and radon due to the lack of adequate fresh air supply. The occupants introduced high levels of CO_2_, which were only removed after extended periods, while the radon concentration decreased primarily due to the occasional opening of doors.

This study highlights the need for more research into effective safety measures to prevent the accumulation of contaminants and the influence of two contaminants on each other. The findings suggest that improvements in ventilation systems are essential to ensure a consistent supply of fresh air, effectively reducing the levels of indoor pollutants.

By distinguishing between human-induced and naturally occurring contaminants, the study provides a foundation for developing targeted strategies to maintain air quality and ensure the safety and well-being of occupants in specialised environments.

Future research should explore integrating advanced ventilation technologies and real-time air quality monitoring systems. These measures could help address air quality issues and ensure a healthier indoor environment. Emphasising occupant behaviour and its impact on indoor air quality can also guide the development of better building management practices and policies.

## Figures and Tables

**Table 1:** Sensors’ range and accuracy.

Measured Parameter	Measurement Range	Measurement Uncertainty
RAC—CR-39 detectors	42 Bq/m^3^ ÷ 16,67 kBq/m^3^	±30%
RAC—AlphaGUARD	2 Bq/m^3^ ÷ 2000 kBq/m^3^	±10%
CO_2_ concentration	0–5000 ppm	±30 ppm
Air temperature	−5 °C ÷ +55 °C	±0.4 °C
Air relative humidity	0% ÷ 90%	±5%

## Data Availability

Data are contained within the article.
